# 5-Benzyl-5*H*-pyrido[3,2-*b*]indole

**DOI:** 10.1107/S1600536811032107

**Published:** 2011-08-17

**Authors:** Julien Letessier, Dieter Schollmeyer, Heiner Detert

**Affiliations:** a University Mainz, Duesbergweg 10-14, 55099 Mainz, Germany

## Abstract

The title compound, C_18_H_14_N_2_, was prepared by twofold Pd-catalyzed aryl­amination of a cyclic pyrido–benzo–iodo­lium salt. In the crystal, two mol­ecules of 9-benzyl-δ-carboline form centrosymmetrical dimers with distances of 3.651 (2) Å between the centroids of the pyridine rings and 3.961 (2) Å between the centroids of the pyrrole and pyridine rings. The phenyl rings point to the other mol­ecule in the dimer and the carboline core is essentially planar [maximum deviation of 0.027 (2) Å].

## Related literature

For δ-Carboline, see: Subbaraju *et al.* (2004 ▶); Paulo *et al.* (2000 ▶); Chernyshev *et al.* (2001 ▶); Namjoshi *et al.* (2011 ▶); Qu *et al.* (2009 ▶); Masterova *et al.* (2008 ▶). For synthetic strategies to carbolines, see: Späth & Eiter (1940 ▶); Sakamoto *et al.* (1999 ▶); Franck *et al.* (2008 ▶). For the transition-metal-catalyzed synthesis of carbazoles, see: Letessier (2011 ▶); Nemkovich *et al.* (2009 ▶). For the transition-metal-catalyzed synthesis of carbolines, see: Nissen *et al.* (2011 ▶), Dassonneville *et al.* (2010 ▶). For β-carboline, see: Torreiles *et al.* (1985 ▶); Love (2006 ▶); Dassonneville *et al.* (2011 ▶); Nissen & Detert (2011 ▶). For the synthesis of the title compound, see: Letessier & Detert (2011 ▶). 
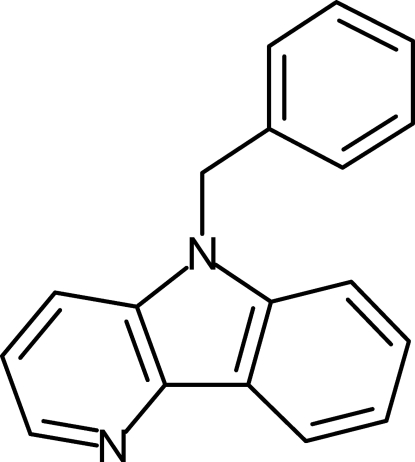


## Experimental

### 

#### Crystal data

C_18_H_14_N_2_
*M* *_r_* = 258.1
Monoclinic, 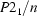

*a* = 11.295 (4) Å
*b* = 10.482 (4) Å
*c* = 11.961 (4) Å
β = 110.387 (11)°
*V* = 1327.4 (8) Å^3^
*Z* = 4
Mo *K*α radiation
μ = 0.08 mm^−1^
*T* = 173 K
0.51 × 0.25 × 0.02 mm

#### Data collection

Bruker SMART CCD diffractometer
15877 measured reflections
3149 independent reflections
1444 reflections with *I* > 2σ(*I*)
*R*_int_ = 0.128

#### Refinement

*R*[*F* ^2^ > 2σ(*F* ^2^)] = 0.055
*wR*(*F* ^2^) = 0.149
*S* = 0.98
3149 reflections
182 parameters
H-atom parameters constrained
Δρ_max_ = 0.22 e Å^−3^
Δρ_min_ = −0.21 e Å^−3^

### 

Data collection: *SMART* (Bruker, 2006 ▶); cell refinement: *SAINT* (Bruker, 2006 ▶); data reduction: *SAINT*; program(s) used to solve structure: *SIR97* (Altomare *et al.*, 1999 ▶); program(s) used to refine structure: *SHELXL97* (Sheldrick, 2008 ▶); molecular graphics: *PLATON* (Spek, 2009 ▶); software used to prepare material for publication: *PLATON*.

## Supplementary Material

Crystal structure: contains datablock(s) I, global. DOI: 10.1107/S1600536811032107/bt5607sup1.cif

Structure factors: contains datablock(s) I. DOI: 10.1107/S1600536811032107/bt5607Isup2.hkl

Supplementary material file. DOI: 10.1107/S1600536811032107/bt5607Isup3.cml

Additional supplementary materials:  crystallographic information; 3D view; checkCIF report

## Figures and Tables

**Fig. 1.**
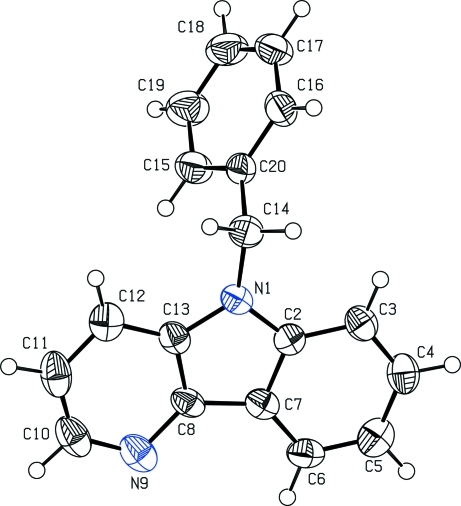
View of compound I. Displacement ellipsoids are drawn at the 50% probability level.

## supplementary crystallographic information

### Comment

The title compound was prepared as a part of a project focused on the transition metal catalyzed synthesis of carbazoles, see: Letessier (2011) and Nemkovich *et al.* (2009), carbolines, see: Nissen, Schollmeyer & Detert (2011) and related indolo-annulated heterocycles, see: Dassonneville *et al.* (2010). Whereas the β-carboline is the core of a large group of alkaloids (see: Torreiles *et al.* (1985); Love (2006)), only a few natural δ-carbolines are known. With Rh- or Ru-catalyzed [2 + 2+2] cycloadditions of alkynyl-ynamides we recently reported a new access to β- and γ-carbolines (Dassonneville *et al.*, 2011), Nissen & Detert (2011), but this approach is not suitable for the synthesis of the δ-isomers. These can now be prepared in a twofold Pd-catalyzed arylation of primary amines with cyclic pyrido-benzo iodolium salts. This unique 9-substituted δ-carboline crystallizes in form of centrosymmetrical dimers. The phenyl group, pointing in the direction of the second molecule, is nearly orthogonal to the essentially planar carboline core (maximal deviations of 0.027 (2) Å from the least square plane). Short distances of the centroid of a pyridine ring of one molecule to the centroid of the pyridine of its counterpart of 3.65 Å and to the pyrrole centroid of 3.96 Å indicate a π-π interaction between the heterocycles.

### Experimental

A solution of 400 mg (0.93 mmol) of benzo[4,5]iodolo[3,2-*b*]pyridin-5-ium trifluoromethanesulfonate (Letessier & Detert, 2011) in dry toluene (10 ml) was deaerated in a Schlenk flask. Under argon, Pd_2_(dba)_3_ (34 mg, 0.04 mmol), Xantphos (64 mg, 0.11 mmol), and Cs_2_CO_3_ (850 mg, 2.61 mmol) were added. The mixture was stirred for 5 min at 300 K before benzyl amine (120 mg, 1.12 mmol) was added. After stirring for 15 h at 383 K, the mixture was cooled to ambient temparature, filtered through celite and concentrated. Purification by column chromatography (petroleum ether / ethyl acetate = 4 / 1) gave 156 mg (65%) of the title compound as colorless crystals with m. p. > 415 K.

### Refinement

Hydrogen atoms attached to carbons were placed at calculated positions with C—H = 0.95 Å (aromatic) or 0.98–0.99 Å (*sp*^3^ C-atom). All H atoms were refined in the riding-model approximation with isotropic displacement parameters (set at 1.2–1.5 times of the *U*_eq_ of the parent atom).

### Crystal data

**Table:**

C_18_H_14_N_2_	*F*(000) = 544
*M**_r_* = 258.1	*D*_x_ = 1.293 Mg m^−^^3^
Monoclinic, *P*2_1_/*n*	Melting point: 415 K
Hall symbol: -P 2yn	Mo *K*α radiation, λ = 0.71073 Å
*a* = 11.295 (4) Å	Cell parameters from 1161 reflections
*b* = 10.482 (4) Å	θ = 2.6–22.3°
*c* = 11.961 (4) Å	µ = 0.08 mm^−^^1^
β = 110.387 (11)°	*T* = 173 K
*V* = 1327.4 (8) Å^3^	Plate, colourless
*Z* = 4	0.51 × 0.25 × 0.02 mm

### Data collection

**Table:**

Bruker SMART CCD diffractometer	1444 reflections with *I* > 2σ(*I*)
Radiation source: sealed Tube	*R*_int_ = 0.128
graphite	θ_max_ = 27.9°, θ_min_ = 2.1°
CCD scan	*h* = −14→14
15877 measured reflections	*k* = −13→13
3149 independent reflections	*l* = −15→15

### Refinement

**Table:**

Refinement on *F*^2^	Secondary atom site location: difference Fourier map
Least-squares matrix: full	Hydrogen site location: inferred from neighbouring sites
*R*[*F*^2^ > 2σ(*F*^2^)] = 0.055	H-atom parameters constrained
*wR*(*F*^2^) = 0.149	*w* = 1/[σ^2^(*F*_o_^2^) + (0.0558*P*)^2^ + 0.2129*P*] where *P* = (*F*_o_^2^ + 2*F*_c_^2^)/3
*S* = 0.98	(Δ/σ)_max_ < 0.001
3149 reflections	Δρ_max_ = 0.22 e Å^−^^3^
182 parameters	Δρ_min_ = −0.21 e Å^−^^3^
0 restraints	Extinction correction: *SHELXL97* (Sheldrick, 2008), Fc^*^=kFc[1+0.001xFc^2^λ^3^/sin(2θ)]^-1/4^
Primary atom site location: structure-invariant direct methods	Extinction coefficient: 0.016 (2)

### Special details

**Table:**

Experimental. ^1^H-NMR (400 MHz, CDCl_3_): δ = 8.59 (dd, J = 4.7 Hz, J = 1.2 Hz, 1H, 2-H), 8.43 (d, J = 7.8 Hz, 1H, 9-H), 7.64 (dd, J = 8.2 Hz, J = 1.2 Hz, 1H, 6-H), 7.53 (td, J = 8.3 Hz, J = 1.2 Hz, 1H, 7-H), 7.42 (d, J = 8.2 Hz, 1H, 6-H), 7.26-7.34 (m, 5H, CH), 7.12 (m, 2H, CH), 5.52 (s, 2H, CH_2_). ^13^C-NMR (75 MHz, CDCl_3_): δ = 141.9 (d, C-2), 141.8 (s, C-9b), 141.4 (s, C-5a), 136.5 (s, C-1), 134.0 (s, C-4a), 128.9 (d, C-2), 127.9 (d, C-7), 127.7 (d, C-4), 126.3 (d, C-3), 122.2 (s, C-9a), 120.9 (d, C-3), 120.1 (d, C-9), 120.0 (d, C-8), 115.8 (d, C-4), 109.2 (d, C-6), 46.5 (t, CH_2_). IR (neat, ATR): ν = 1621 (w), 1588 (w), 1482 (m), 1451 (m), 1412 (s), 1334 (m), 1318 (s), 1242 (w), 1193 (m), 1115 (w), 1012 (w), 913 (w), 845 (m), 781 (s), 742 (vs), 730 (vs), 721 (vs), 695 (s)cm^-1^. FD-MS: m/z = 258.1 [C_18_H_14_N_2_]^+^.
Geometry. All e.s.d.'s (except the e.s.d. in the dihedral angle between two l.s. planes) are estimated using the full covariance matrix. The cell e.s.d.'s are taken into account individually in the estimation of e.s.d.'s in distances, angles and torsion angles; correlations between e.s.d.'s in cell parameters are only used when they are defined by crystal symmetry. An approximate (isotropic) treatment of cell e.s.d.'s is used for estimating e.s.d.'s involving l.s. planes.
Refinement. Refinement of *F*^2^ against ALL reflections. The weighted *R*-factor *wR* and goodness of fit *S* are based on *F*^2^, conventional *R*-factors *R* are based on *F*, with *F* set to zero for negative *F*^2^. The threshold expression of *F*^2^ > σ(*F*^2^) is used only for calculating *R*-factors(gt) *etc*. and is not relevant to the choice of reflections for refinement. *R*-factors based on *F*^2^ are statistically about twice as large as those based on *F*, and *R*- factors based on ALL data will be even larger.

### Fractional atomic coordinates and isotropic or equivalent isotropic displacement parameters (Å^2^)

**Table:**

	*x*	*y*	*z*	*U*_iso_*/*U*_eq_	
N1	0.74211 (17)	0.47469 (18)	0.17200 (17)	0.0360 (5)	
C2	0.7372 (2)	0.4852 (2)	0.2855 (2)	0.0334 (6)	
C3	0.8208 (2)	0.5471 (2)	0.3853 (2)	0.0410 (6)	
H3	0.8945	0.5885	0.3825	0.049*	
C4	0.7916 (3)	0.5456 (2)	0.4876 (2)	0.0468 (7)	
H4	0.8463	0.5873	0.5568	0.056*	
C5	0.6848 (3)	0.4849 (2)	0.4923 (2)	0.0501 (7)	
H5	0.6678	0.4861	0.5647	0.060*	
C6	0.6034 (2)	0.4233 (2)	0.3955 (2)	0.0424 (7)	
H6	0.5304	0.3817	0.3998	0.051*	
C7	0.6295 (2)	0.4231 (2)	0.2914 (2)	0.0356 (6)	
C8	0.5662 (2)	0.3720 (2)	0.1737 (2)	0.0365 (6)	
N9	0.45662 (19)	0.3047 (2)	0.1336 (2)	0.0488 (6)	
C10	0.4201 (2)	0.2725 (3)	0.0174 (3)	0.0514 (8)	
H10	0.3436	0.2259	−0.0154	0.062*	
C11	0.4853 (3)	0.3021 (2)	−0.0574 (2)	0.0522 (8)	
H11	0.4528	0.2755	−0.1384	0.063*	
C12	0.5981 (2)	0.3703 (2)	−0.0164 (2)	0.0452 (7)	
H12	0.6447	0.3914	−0.0664	0.054*	
C13	0.6378 (2)	0.4055 (2)	0.1036 (2)	0.0370 (6)	
C14	0.8358 (2)	0.5335 (2)	0.1306 (2)	0.0409 (6)	
H14A	0.8610	0.6162	0.1719	0.049*	
H14B	0.7963	0.5513	0.0442	0.049*	
C15	0.9524 (2)	0.4548 (2)	0.1502 (2)	0.0360 (6)	
C16	1.0688 (2)	0.5125 (3)	0.1743 (2)	0.0475 (7)	
H16	1.0757	0.6027	0.1820	0.057*	
C17	1.1752 (2)	0.4406 (3)	0.1872 (2)	0.0548 (8)	
H17	1.2544	0.4817	0.2035	0.066*	
C18	1.1675 (3)	0.3100 (3)	0.1769 (2)	0.0583 (8)	
H18	1.2407	0.2606	0.1857	0.070*	
C19	1.0529 (3)	0.2523 (3)	0.1537 (3)	0.0606 (9)	
H19	1.0468	0.1621	0.1471	0.073*	
C20	0.9462 (2)	0.3233 (3)	0.1399 (2)	0.0487 (7)	
H20	0.8673	0.2815	0.1232	0.058*	

### Atomic displacement parameters (Å^2^)

**Table:**

	*U*^11^	*U*^22^	*U*^33^	*U*^12^	*U*^13^	*U*^23^
N1	0.0301 (11)	0.0362 (12)	0.0403 (13)	−0.0008 (9)	0.0106 (9)	0.0009 (9)
C2	0.0340 (13)	0.0264 (13)	0.0380 (15)	0.0064 (11)	0.0102 (11)	0.0032 (11)
C3	0.0415 (14)	0.0330 (14)	0.0454 (17)	−0.0039 (12)	0.0113 (12)	0.0013 (12)
C4	0.0553 (17)	0.0366 (16)	0.0437 (17)	−0.0044 (14)	0.0113 (13)	−0.0011 (13)
C5	0.0628 (19)	0.0421 (16)	0.0487 (17)	0.0031 (15)	0.0234 (15)	0.0025 (14)
C6	0.0381 (15)	0.0372 (15)	0.0577 (19)	0.0002 (12)	0.0239 (14)	0.0030 (13)
C7	0.0313 (13)	0.0277 (13)	0.0459 (16)	0.0049 (11)	0.0112 (12)	0.0052 (11)
C8	0.0263 (12)	0.0303 (14)	0.0499 (16)	0.0017 (11)	0.0097 (11)	0.0029 (12)
N9	0.0354 (12)	0.0359 (13)	0.0652 (16)	0.0046 (11)	0.0050 (11)	0.0021 (12)
C10	0.0363 (15)	0.0358 (16)	0.066 (2)	0.0038 (12)	−0.0024 (15)	−0.0015 (14)
C11	0.0535 (18)	0.0396 (17)	0.0447 (17)	0.0077 (15)	−0.0066 (14)	−0.0034 (13)
C12	0.0494 (16)	0.0372 (15)	0.0442 (17)	0.0081 (13)	0.0102 (13)	0.0032 (12)
C13	0.0295 (13)	0.0314 (14)	0.0440 (16)	0.0058 (11)	0.0050 (12)	−0.0006 (12)
C14	0.0408 (14)	0.0394 (15)	0.0438 (16)	−0.0017 (12)	0.0161 (12)	0.0034 (12)
C15	0.0353 (14)	0.0397 (15)	0.0324 (14)	−0.0023 (12)	0.0112 (11)	−0.0013 (11)
C16	0.0444 (16)	0.0535 (17)	0.0422 (16)	−0.0130 (14)	0.0123 (13)	−0.0050 (13)
C17	0.0344 (15)	0.081 (2)	0.0485 (18)	−0.0076 (16)	0.0140 (13)	−0.0022 (16)
C18	0.0416 (17)	0.075 (2)	0.061 (2)	0.0133 (17)	0.0214 (14)	0.0001 (17)
C19	0.0534 (19)	0.0505 (19)	0.084 (2)	0.0045 (15)	0.0321 (17)	−0.0043 (16)
C20	0.0379 (15)	0.0436 (16)	0.0663 (19)	−0.0008 (13)	0.0202 (13)	−0.0050 (14)

### Geometric parameters (Å, °)

**Table:**

N1—C2	1.382 (3)	C11—C12	1.393 (4)
N1—C13	1.383 (3)	C11—H11	0.9500
N1—C14	1.453 (3)	C12—C13	1.395 (3)
C2—C3	1.397 (3)	C12—H12	0.9500
C2—C7	1.403 (3)	C14—C15	1.502 (3)
C3—C4	1.373 (3)	C14—H14A	0.9900
C3—H3	0.9500	C14—H14B	0.9900
C4—C5	1.382 (4)	C15—C16	1.383 (3)
C4—H4	0.9500	C15—C20	1.384 (3)
C5—C6	1.365 (3)	C16—C17	1.380 (4)
C5—H5	0.9500	C16—H16	0.9500
C6—C7	1.376 (3)	C17—C18	1.374 (4)
C6—H6	0.9500	C17—H17	0.9500
C7—C8	1.442 (3)	C18—C19	1.366 (4)
C8—N9	1.358 (3)	C18—H18	0.9500
C8—C13	1.398 (3)	C19—C20	1.376 (4)
N9—C10	1.348 (3)	C19—H19	0.9500
C10—C11	1.378 (4)	C20—H20	0.9500
C10—H10	0.9500		
			
C2—N1—C13	107.80 (19)	C11—C12—C13	115.1 (3)
C2—N1—C14	125.75 (19)	C11—C12—H12	122.4
C13—N1—C14	126.3 (2)	C13—C12—H12	122.4
N1—C2—C3	129.1 (2)	N1—C13—C12	130.5 (2)
N1—C2—C7	110.2 (2)	N1—C13—C8	109.2 (2)
C3—C2—C7	120.8 (2)	C12—C13—C8	120.3 (2)
C4—C3—C2	117.1 (2)	N1—C14—C15	114.7 (2)
C4—C3—H3	121.4	N1—C14—H14A	108.6
C2—C3—H3	121.4	C15—C14—H14A	108.6
C3—C4—C5	121.7 (2)	N1—C14—H14B	108.6
C3—C4—H4	119.1	C15—C14—H14B	108.6
C5—C4—H4	119.1	H14A—C14—H14B	107.6
C6—C5—C4	121.5 (3)	C16—C15—C20	118.0 (2)
C6—C5—H5	119.2	C16—C15—C14	120.7 (2)
C4—C5—H5	119.2	C20—C15—C14	121.2 (2)
C5—C6—C7	118.4 (2)	C17—C16—C15	120.7 (3)
C5—C6—H6	120.8	C17—C16—H16	119.6
C7—C6—H6	120.8	C15—C16—H16	119.6
C6—C7—C2	120.5 (2)	C18—C17—C16	120.6 (3)
C6—C7—C8	133.9 (2)	C18—C17—H17	119.7
C2—C7—C8	105.5 (2)	C16—C17—H17	119.7
N9—C8—C13	124.4 (2)	C19—C18—C17	119.0 (3)
N9—C8—C7	128.3 (2)	C19—C18—H18	120.5
C13—C8—C7	107.4 (2)	C17—C18—H18	120.5
C10—N9—C8	114.1 (2)	C18—C19—C20	120.9 (3)
N9—C10—C11	124.9 (3)	C18—C19—H19	119.6
N9—C10—H10	117.5	C20—C19—H19	119.6
C11—C10—H10	117.5	C19—C20—C15	120.8 (3)
C10—C11—C12	121.1 (3)	C19—C20—H20	119.6
C10—C11—H11	119.4	C15—C20—H20	119.6
C12—C11—H11	119.4		
			
C13—N1—C2—C3	−179.4 (2)	C10—C11—C12—C13	−0.3 (4)
C14—N1—C2—C3	−3.2 (4)	C2—N1—C13—C12	178.9 (2)
C13—N1—C2—C7	−0.1 (2)	C14—N1—C13—C12	2.8 (4)
C14—N1—C2—C7	176.1 (2)	C2—N1—C13—C8	0.1 (2)
N1—C2—C3—C4	178.4 (2)	C14—N1—C13—C8	−176.1 (2)
C7—C2—C3—C4	−0.8 (3)	C11—C12—C13—N1	−178.6 (2)
C2—C3—C4—C5	0.4 (4)	C11—C12—C13—C8	0.2 (3)
C3—C4—C5—C6	0.2 (4)	N9—C8—C13—N1	179.3 (2)
C4—C5—C6—C7	−0.3 (4)	C7—C8—C13—N1	0.0 (3)
C5—C6—C7—C2	−0.2 (4)	N9—C8—C13—C12	0.3 (4)
C5—C6—C7—C8	−178.6 (2)	C7—C8—C13—C12	−179.0 (2)
N1—C2—C7—C6	−178.6 (2)	C2—N1—C14—C15	88.4 (3)
C3—C2—C7—C6	0.7 (4)	C13—N1—C14—C15	−96.1 (3)
N1—C2—C7—C8	0.1 (2)	N1—C14—C15—C16	−146.8 (2)
C3—C2—C7—C8	179.5 (2)	N1—C14—C15—C20	35.9 (3)
C6—C7—C8—N9	−0.8 (4)	C20—C15—C16—C17	0.2 (4)
C2—C7—C8—N9	−179.3 (2)	C14—C15—C16—C17	−177.2 (2)
C6—C7—C8—C13	178.4 (3)	C15—C16—C17—C18	−0.3 (4)
C2—C7—C8—C13	−0.1 (2)	C16—C17—C18—C19	−0.1 (4)
C13—C8—N9—C10	−0.6 (3)	C17—C18—C19—C20	0.5 (4)
C7—C8—N9—C10	178.5 (2)	C18—C19—C20—C15	−0.5 (4)
C8—N9—C10—C11	0.5 (4)	C16—C15—C20—C19	0.2 (4)
N9—C10—C11—C12	−0.1 (4)	C14—C15—C20—C19	177.6 (2)
